# Deep generative models in DataSHIELD

**DOI:** 10.1186/s12874-021-01237-6

**Published:** 2021-04-03

**Authors:** Stefan Lenz, Moritz Hess, Harald Binder

**Affiliations:** Institute of Medical Biometry and Statistics, Faculty of Medicine and Medical Center – University of Freiburg, Freiburg, Germany

**Keywords:** Privacy/statistics and numerical data, Biomedical research/methods, Deep learning, Distributed system

## Abstract

**Background:**

The best way to calculate statistics from medical data is to use the data of individual patients. In some settings, this data is difficult to obtain due to privacy restrictions. In Germany, for example, it is not possible to pool routine data from different hospitals for research purposes without the consent of the patients.

**Methods:**

The DataSHIELD software provides an infrastructure and a set of statistical methods for joint, privacy-preserving analyses of distributed data. The contained algorithms are reformulated to work with aggregated data from the participating sites instead of the individual data. If a desired algorithm is not implemented in DataSHIELD or cannot be reformulated in such a way, using artificial data is an alternative. Generating artificial data is possible using so-called generative models, which are able to capture the distribution of given data. Here, we employ deep Boltzmann machines (DBMs) as generative models. For the implementation, we use the package “BoltzmannMachines” from the Julia programming language and wrap it for use with DataSHIELD, which is based on R.

**Results:**

We present a methodology together with a software implementation that builds on DataSHIELD to create artificial data that preserve complex patterns from distributed individual patient data. Such data sets of artificial patients, which are not linked to real patients, can then be used for joint analyses. As an exemplary application, we conduct a distributed analysis with DBMs on a synthetic data set, which simulates genetic variant data. Patterns from the original data can be recovered in the artificial data using hierarchical clustering of the virtual patients, demonstrating the feasibility of the approach. Additionally, we compare DBMs, variational autoencoders, generative adversarial networks, and multivariate imputation as generative approaches by assessing the utility and disclosure of synthetic data generated from real genetic variant data in a distributed setting with data of a small sample size.

**Conclusions:**

Our implementation adds to DataSHIELD the ability to generate artificial data that can be used for various analyses, e.g., for pattern recognition with deep learning. This also demonstrates more generally how DataSHIELD can be flexibly extended with advanced algorithms from languages other than R.

## Background

In large consortia, pooling of individual level data is often not possible due to data security and data protection concerns. Thus, techniques for distributed privacy-preserving analysis are needed. For example, the MIRACUM consortium [1], a joint project of ten university hospitals in Germany, aims to show how patient data that are distributed across sites can be jointly analysed. In this consortium, a particular goal is to apply advanced machine learning techniques for identifying complex interaction patterns in medical data.

One general way to enable such analysis techniques on distributed data is to use a synthetic data approach. Synthetic datasets mimic statistical features of the original data without any linkage to individuals in the original data. These synthetic data can then be shared across the sites for joint analyses. For simple statistical analyses, this approach has been found to work well [2–4], and there are even commercial offerings for business data [5]. Multivariable statistical analyses are also feasible. For example, an approach using bivariate copulas can recreate complex marginal distributions and provide results similar to the original data when using multivariable linear mixed regression for analysis [6].

However, it is still not fully resolved how to create synthetic data that also reflect complex patterns, which might then, e.g., be analysed using machine learning tools. This may require more complex approaches to generate the synthetic data. Generative adversarial networks (GANs) [7], among other generative approaches [8], have been proposed as a solution. In particular, generative deep learning approaches might be useful, as they can represent complex patterns [9] and have been shown to be feasible for small sample sizes [10, 11]. Correspondingly, we decided to develop an implementation for artificial data based on deep learning within the DataSHIELD framework for distributed analysis. DataSHIELD [12] is a software tool used in many multicentre studies for distributed privacy-preserving analysis, and which offers many statistical tools for researchers. Its implementation is based on meta-analysis techniques or parameter estimation via distributed calculation. A synthetic data approach in DataSHIELD will thus provide even more flexible data analysis tools to an already very active user community.

In the following, we present the implementation of our approach using deep Boltzmann machines (DBMs) [13] as generative models in DataSHIELD. Deep Boltzmann machines were chosen as generative models for synthetic data on the basis of their good performance on data sets with small sample sizes, also compared to variational autoencoders (VAEs) and GANs [10, 11]. This is of particular importance, e.g., when the overall sample size is moderate, but sample size per site is small. We implemented the algorithms for fitting DBMs in the Julia programming language [14], which is, compared to R, better suited for implementing deep learning algorithms. To access the algorithms in DataSHIELD, we integrated them in the statistical analysis environment R [15], which is the basis for DataSHIELD, via a package for interfacing Julia and R.

We demonstrate our approach based on genetic variant data, specifically single nucleotide polymorphism (SNP) data. Similarly to electronic patient records, these contain highly sensitive information about individuals, and are therefore particularly interesting for distributed privacy-preserving analyses. We also show the feasibility of the approach with empirical studies investigating different numbers of sites and sample sizes per site in a distributed analysis, and compare our approach to other types of generative models, namely GANs, VAEs, and additionally multivariate imputation by chained equations (MICE).

## Methods

### DataSHIELD

DataSHIELD is open-source software that is already used in the field of epidemiology for the analysis of multi-centre cohort studies. Analyses in DataSHIELD are performed without individual data leaving the sites. This is possible by using reformulated algorithms that solely rely on aggregated statistics. Only those aggregated statistics leave the sites and are used to calculate the final result. In this way, the DataSHIELD software allows users to perform several types of descriptive statistics and standard statistical models. For example, it is possible to compute linear regression models via DataSHIELD on data sets that are distributed among several sites, and get the same results as with pooled data. The user can access the DataSHIELD functionality by using functions of specific packages in the R programming language [15].

The Opal web server software [16], running in separate instances at each of the sites participating in a federated analysis, provides the decentralised data. Its interface is secured by authentication and authorization. Some users may have the right to view data, while others may only access aggregated data by calling specific R functions that are approved by the organisation operating the Opal instance. These R functions, most of which are collected in packages specifically for use in DataSHIELD, must only return data that does not disclose information about individuals. The official DataSHIELD packages are designed and reviewed specifically to minimise the disclosure risk. In addition to the existing package ecosystem, the infrastructure is extensible and allows developers to write their own R packages, which then can be installed by administrators of Opal instances.

### Deep Boltzmann machines (DBMs) as generative models

The goal of generative models is to capture the probability distribution of multiple variables in a model, allowing new samples to be drawn from the model according to this distribution. Generative models are trained in an unsupervised manner with data from the original distribution as input. In many cases, they can also be used to find higher-level representations of the data [9] by analysing the model parameters.

Here, we will focus on deep Boltzmann machines as generative models. General Boltzmann machines are stochastic neural networks whose nodes have an activation probability *p*(*v*, *h*) that is determined by the energy function *E* of the network.

$$ p\left(v,h\right)=\frac{e^{-E\left(v,h\right)}}{Z}\ \mathsf{with}\ Z=\sum \limits_{v,h}{e}^{-E\left(v,h\right)} $$

Thus, Boltzmann machines are so-called “energy-based” models. The nodes are divided into two groups. The visible nodes (*v*) receive the data input, while the hidden nodes (*h*) encode latent variables of the data. The normalization constant *Z* is also called the *partition function.* Due to the large number of terms in the sum for *Z*, which runs over all possible configurations of activations of nodes, computing the real value of the probability is too complex for most use cases. In practice, Gibbs sampling is used instead to sample from the model. With Gibbs sampling, it is also easy to sample conditionally on specific variables, which makes it possible to use Boltzmann machines as generative models in “what-if” scenarios. For example, in a medical setting, a Boltzmann machine trained on patients’ diagnoses can be used to generate synthetic patient data with specific disease patterns, even if these patterns are relatively rare in the original data. A use case for this may be to simulate a population of patients for planning a new study.

**Fig. 1:**
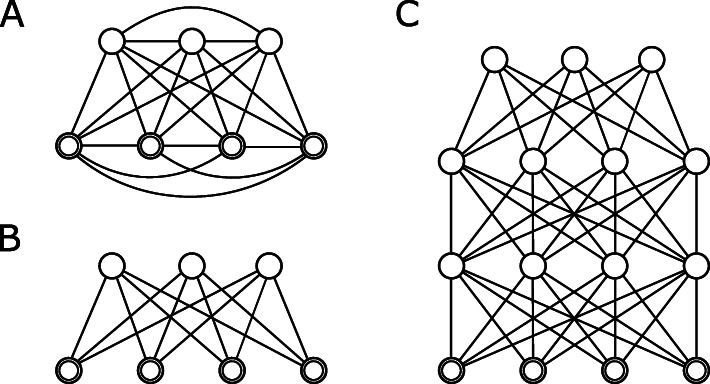
Overview of different types of Boltzmann machines. The visible nodes are depicted as doubled circles, hidden nodes are single circles. a: General Boltzmann machine, with all nodes connected to each other. b: Restricted Boltzmann machines, with two layers of nodes. c: Deep belief network (DBN) or deep Boltzmann machine (DBM), consisting of multiple layers. The architecture of DBNs and DBMs is the same but the algorithms for training and sampling are different

The network of general Boltzmann machines is a complete undirected graph, where all nodes are connected to each other (see Fig. 1 a). A first step in making Boltzmann machines practically usable was to use *restricted Boltzmann machines* (RBMs). These restrict the connections in the graph, disallowing connections between visible nodes as well as connections between hidden nodes. Thereby, the graph of the network forms a complete bipartite graph that partitions the set of vertices into the set of visible nodes, the *visible layer*, and the set of hidden nodes, the *hidden layer* (see Fig. 1 b). This allows for the rapid calculation of the conditional probabilities because in this case it is possible to derive simple formulas for the conditional probabilities, which can be calculated for all nodes in a layer in a vectorised way. This is the basis for an effective training algorithm for RBMs called *contrastive divergence* [17]. RBMs can also be used as generative models but the restrictions on the connections in the network also limit their power to model distributions.

Subsequently, it was discovered that stacking restricted Boltzmann machines on top of each other (see Fig. 1 c) by training the next restricted Boltzmann machines with the hidden activations of the previous one enabled the networks to learn features of increasing abstraction. The resulting model is a *deep belief network* (DBN), and the training procedure is called greedy layer-wise training [18]. Although their architecture is well suited for dimension reduction, DBNs are less powerful than the next stage of development, the *deep Boltzmann machines* (DBMs). Deep belief networks can be used as generative models by sampling in the last restricted Boltzmann machine and then using the conditional probabilities to propagate the activation to the visible nodes. This way, the full information of the network is not harnessed equally to generate new samples. Deep Boltzmann machines, on the other hand, have the same network layout as deep belief networks, but generate samples employing the full network. This is similar to a general Boltzmann machine, albeit restricted to a layered layout. For training, DBMs are optimised with an algorithm for maximising the variational lower bound of the likelihood in the Boltzmann machine model. This algorithm is also referred to as *fine-tuning* because greedy layer-wise pre-training is carried out to provide a good starting point for the variational likelihood algorithm, which would otherwise not succeed in finding a good local optimum.

Due to the intractable nature of the partition function, monitoring the optimisation process and evaluating the resulting model is difficult. For restricted Boltzmann machines, the *reconstruction error* is a proxy measure that follows the likelihood of the model very well. It is calculated by taking the data as activation of the visible nodes, calculating the probabilities for the activations in the hidden layer conditioned on the data input and then again calculating the probabilities of the visible layer conditioned on the hidden probabilities. This last result is called the reconstruction of the data input. The reconstruction error is the distance to the original data. It can also be used to monitor the greedy layer-wise training of deep belief networks and for the pre-training of deep Boltzmann machines.

With a stochastic algorithm called *annealed importance sampling* (AIS), it is possible to estimate the likelihood of restricted and deep Boltzmann machines [19]. It is needed in particular to measure the training objective of DBMs, the variational lower bound of the likelihood.

We employ a package for the Julia programming language [14] that implements these algorithms and provides a user-friendly interface for training and evaluating deep Boltzmann machines [20]. Further on, we show how we integrate this with the DataSHIELD software and concept.

### Implementation of deep Boltzmann machines in DataSHIELD

**Fig. 2:**
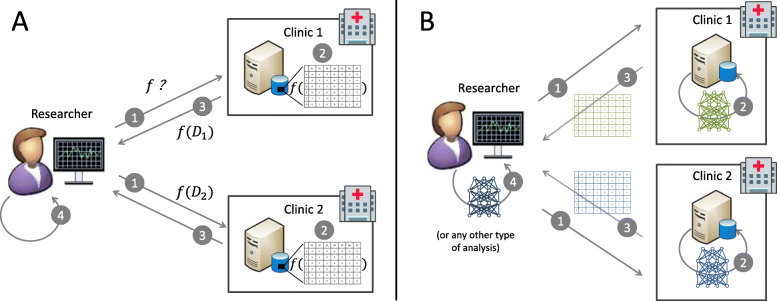
Applying the DataSHIELD principle in working with synthetic data from generative models. The standard DataSHIELD approach is depicted in panel a: The researcher sends a request via the DataSHIELD infrastructure (1). The sites then calculate aggregated statistics (2) and return them to the researcher (3). These statistics do not allow conclusions about individual patients, but can be used to derive useful information about the population (4). When working with generated models and synthetic data (panel b), the workflow is similar. The researcher requests the training of a generative model (1). Once the model has been trained on the server side with access to the individual-level data (2), synthetic samples can be generated (3). The researcher can use the synthetic data to conduct further analyses (4)

The developed software allows users to remotely train deep Boltzmann machines without requiring the users to have access to the individual-level data. Trained models can be used, e.g., to generate synthetic data as depicted in Fig. 2.

We provide our implementation of DBMs in DataSHIELD as open-source software. It consists of a client-side R package [21] and a server-side R package [22] that can be installed in an Opal server and called from the client-side. The server-side needs Julia with the package “BoltzmannMachines” installed in addition. The functionality of the “BoltzmannMachines” Julia package is imported into R via the “JuliaConnectoR” R package [23]. This provides a generic interface that allows the use of Julia functions in R and thereby makes it possible to obtain the speed advantages of the Julia code while using the DataSHIELD R interface.

According to the design principle of DataSHIELD, the parts of the algorithms that need access to individual-level data are executed on the server-side, where the data is stored, and only aggregated data leaves site. Our approach follows this principle. The training procedure for the DBMs, which needs access to individual-level data, is performed on the server-side. Only the generated synthetic data and information for monitoring the training success are communicated with the client.

From a technical perspective, it is straightforward to also transfer the models themselves outside via DataSHIELD, because the DataSHIELD infrastructure can transfer arbitrary R objects to the client. It is the responsibility of the developers of DataSHIELD functionality to ensure that the returned values do not disclose sensitive information about individuals. For basic aggregated statistics it is possible to prove mathematically how much information about individuals is contained. For neural networks, this is very hard because they are so complex and usually consist of a very high number of parameters. In many cases, the number of parameters is even higher than the number of data points in the training data itself. Thus, it is very hard to prove that a neural network cannot be hacked. Model inversion attacks, which aim to extract information about individual data sets from trained models, are being researched and developed [24]. Therefore, we do not allow the transfer of the models by default, but give data custodians the option to explicitly allow this in the Opal server environment if there is enough trust in the given setting.

**Fig. 3:**
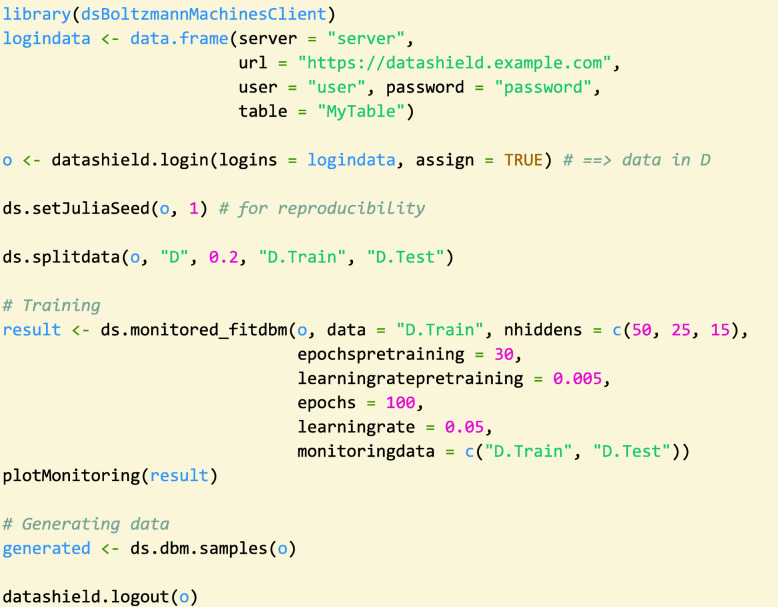
Example code for training a deep Boltzmann machine and using it as a generative model. First, the user needs to log in to the Opal server, where the data is stored. If the specified data set is available, and the user has the correct access rights, the data set is loaded into the R session. The loaded data can be split into training and test data before the training. In the subsequent call to the fitting function, which by default also collects monitoring data from the training, the most important parameters for training a DBM are included. The numbers of hidden nodes for each of the hidden layers (“nhiddens”) determine the model architecture. The learning rate and the number of epochs for pre-training and fine-tuning of the DBM are the most important parameters for the optimisation procedure. If a good solution has been found, the model can be used to generate synthetic data and return it to the client

An additional challenge, common to all neural networks, is the extensive hyperparameter tuning that the training requires. Table 1 gives an overview of the tuning parameters for training a DBM (see function “ds.monitored_fitdbm” in Table 2). As shown in Fig. 3, the number of epochs and the learning rate, together with the model architecture, are parameters that are highly important for successful training. These parameters must be tuned individually for different data sets, as the learning rate depends on how informative the different samples are, and the number of epochs must be adjusted accordingly. The architecture must be deep enough to be able to capture the important structure. At the same time, the model should not have too many parameters to avoid overfitting and computational costs. To choose these parameters, our software provides different metrics to assess the model quality during and after the training. It offers functions to estimate the likelihood (for RBMs and DBMs) and the lower bound of the likelihood (for DBMs) via AIS. For smaller models, it is also possible to calculate the likelihood exactly. These evaluations can be collected during training to monitor its success. The monitoring output can be transferred and displayed to the DataSHIELD client without privacy issues, even if the number of training attempts is high, because it does not contain information about individual patient data. In this way, the user can see the performance and select good hyperparameters without having direct access to the models. After a successful training, the final model can then be used to generate synthetic data that is handed to the researcher.

**Table 1:** Most important hyperparameters for fitting a DBM. These parameters can be specified in the function “ds.monitored_fitdbm” (see Table 2). The parameters for pre-training can also be controlled individually for each layer (i.e. for each RBM in the stack) via the function “ds.bm.defineLayer”. Together with the function “ds.bm.definePartitionedLayer”, this allows to also create models with partitioned architectures

Hyperparameter name	Meaning of hyperparameter
learningrate	Learning rate for stochastic gradient descent optimization
learningratepretraining	Learning rate for pre-training, may be specified separately
epochs	Number of training epochs
epochspretraining	Number of epochs for pre-training, may be specified separately
nhiddens	Number of hidden nodes specified as a vector of numbers, containing one number for each hidden layer
batchsizepretraining	Batch size used in pre-training

**Table 2:** Overview of client-side functions for training and using DBM models

Function name	Short description
ds.monitored_fitrbm	Monitored training of an RBM model
ds.monitored_stackrbms	Monitored training of a stack of RBMs. Can be used for pre-training a DBM or for training a DBN
ds.monitored_fitdbm	Monitored training of a DBM, including pre-training and fine-tuning
ds.setJuliaSeed	Set a seed for the random number generator
ds.dbm.samples/ ds.rbm.samples	Generate samples from a DBM/RBM. This also allows conditional sampling.
ds.bm.defineLayer	Define training parameters individually for a RBM layer in a DBM or DBN
ds.bm.definePartitionedLayer	Define a partitioned layer using other layers as parts
ds.dbm.top2LatentDims	Get a two-dimensional representation of latent features
ds.rbm.loglikelihood	Estimates the partition function of an RBM with AIS and then calculates the log-likelihood
ds.dbm.loglikelihood	Performs a separate AIS run for each of the samples to estimate the log-likelihood of a DBM
ds.dbm.logproblowerbound	Estimates the variational lower bound of the likelihood of a DBM with AIS
ds.rbm.exactloglikelihood/ ds.dbm.exactloglikelihood	Calculates the log-likelihood for a RBM/DBM (exponential complexity)

**Table 3:** Results for the comparison of different distributed generative models

**RMSE of log odds ratios** ***d*(*x***_***gen***_, ***x***_***val***_**): Median (5% quantile - 95% quantile)**				
	**1 site**	**2 sites**	**5 sites**	**20 sites**
DBM	0.98 (0.82 – 1.21)	1.00 (0.78 – 1.19)	1.03 (0.74 – 1.65)	1.42 (0.89 – 3.85)
GAN	2.17 (1.86 – 3.36)	3.28 (2.12 – 6.33)	3.82 (2.41 – 6.41)	4.00 (2.41 – 6.88)
IM	3.96 (2.33 – 6.95)	3.95 (2.32 – 6.95)	3.97 (2.32 – 6.95)	3.90 (2.30 – 6.84)
MICE	3.72 (2.87 – 6.04)	3.41 (2.76 – 5.13)	3.05 (2.47 – 4.61)	2.84 (1.93 – 4.22)
VAE	1.05 (0.72 – 1.85)	1.02 (0.68 – 1.72)	0.90 (0.61 – 1.50)	0.89 (0.53 – 1.53)
**Proportion of overfitting: Median (5% quantile - 95% quantile)**				
	**1 site**	**2 sites**	**5 sites**	**20 sites**
DBM	0.11 (-0.016 – 0.24)	-0.14 (-0.44 – 0.27)	-0.15 (-0.39 – 0.24)	0.38 (0.11 – 0.69)
GAN	0.068 (0.013 – 0.17)	0.12 (0.018 – 0.31)	0.22 (0.15 – 0.34)	0.4 (0.29 – 0.55)
IM	0.055 (-0.0045 – 0.099)	0.12 (0.058 – 0.18)	0.22 (0.15 – 0.31)	0.45 (0.30 – 0.63)
MICE	0.031 (0.0022 – 0.08)	0.10 (0.0048 – 0.32)	0.18 (-0.019 – 0.45)	0.45 (0.09 – 0.85)
VAE	0.19 (0.057 – 0.32)	0.27 (0.062 – 0.45)	0.29 (0.064 – 0.56)	0.48 (-0.20 – 0.82)

### Benchmarking of generative performance

For quantifying the quality of samples, i.e. how well these match the target distribution, Goncalves et al. [8] proposed to use the Euclidean distance of the pair-wise correlation matrices computed from original observations and generated synthetic data. Here, we employ a similar approach but use odds ratios instead of correlations. Odds ratios indicate the pair-wise association of binary variables and are directly related to the probability of observing a state for a variable conditional on the state of another variable. Since we study binary SNP data, compared to correlations, odds ratios can be interpreted more easily in this scenario. Further, odds ratios are closely linked to logistic regression as a standard analysis technique.

We evaluate the generative performance of the models similar to our approach in Nußberger et al. [11]. Specifically, we compute the matrices of pair-wise odds-ratios for all variables separately for a validation data set *x*_*val*_ and a generated data set *x*_*gen*_. (To get finite values for the odds ratios in all cases, zeros in cross tables are replaced with the value 0.5.) We then compute the logarithm (log) of the odds ratios. Since the resulting matrices of log odds ratios are symmetric, we only use the lower half of the matrices to compute the value *d*(*x*_*gen*_, *x*_*val*_) as the rooted mean squared error (RMSE) between the resulting log odds ratios in generated and original data. The resulting value of *d*(*x*_*gen*_, *x*_*val*_) indicates the distance between a generated data set and a validation data set based on the pair-wise odds ratios in the respective data sets.

### Measuring disclosure

Similarly to Goncalves et al. [8], we want to compare the models not only by measuring their performance but also with respect to privacy. Here, we want to consider membership privacy in particular. This privacy notion stems from privacy breaches where attackers could identify individuals in published, supposedly anonymized data sets [25]. Metrics for membership privacy measure the amount of information about individuals from the input data set that is contained in a model or in the output of a model. Differential privacy is a special type of these metrics. Intuitively, an algorithm satisfies differential privacy with some pre-specified boundary *ε*, if a single input vector does not change the result more than it is allowed by that boundary. This can be achieved by adding noise and clipping the gradients in the optimisation procedure of neural networks [26]. This, of course, reduces the quality of the resulting model fit. Another problem of differential privacy, which has not been solved yet, is a standardised way for determining a value for the parameter *ε* that is sufficient for, e. g., applications in health care [27]. Instead, we use simpler, alternative disclosure metrics for measuring membership privacy here.

One approach for this is to take a look at the amount of overfitting in the model, i.e., the extent to which a model captures the information about the individuals used for training better than the information about the general underlying structure. For measuring this extent, we define the proportion of overfitting as

1 $$ \frac{\boldsymbol{d}\left({\boldsymbol{x}}_{\boldsymbol{gen}},{\boldsymbol{x}}_{\boldsymbol{val}}\right)-\boldsymbol{d}\left({\boldsymbol{x}}_{\boldsymbol{gen}},{\boldsymbol{x}}_{\boldsymbol{train}}\right)}{\boldsymbol{d}\left({\boldsymbol{x}}_{\boldsymbol{gen}},{\boldsymbol{x}}_{\boldsymbol{val}}\right)}. $$

The respective data sets are the training data set *x*_*train*_, the validation dataset *x*_*val*_, and the set of generated data *x*_*gen*_. The proportion of overfitting indicates how well the model has learned the training data compared to the validation data. The higher it is, the more the model has learned about specific details of the training data rather than about the underlying structure of the data. If the model learns too much about the specifics of the training data set, the probability of disclosure of information about individuals contained therein increases. If, however, the model fit is equally good on training and validation data, the model has only captured more general information of the data that is not tied to a particular collection of individuals. Therefore, the proportion of overfitting can be used as a rough metric to compare the different generative models with respect to membership disclosure.

Additionally, we simulate a form of a membership attack [8, 28] for measuring the disclosure risk. For this simulation we use a training data set, a test data set of the same size as the training data set, and a generated data set. The attacker guesses whether a certain given sample is a training sample by whether there is a generated sample in a pre-specified distance of the given sample. The distance between samples is measured here as the absolute-value norm, which is equivalent to the Hamming distance for binary vectors. For each sample in the training data set, the attacker may guess right (true positive) or wrong (false negative). Analogously, for each sample in the test data set, the attacker may guess right (true negative) or wrong (false positive). We summarize the success of the attacker by reporting precision and sensitivity of the guesses that are performed with each sample in test and training data. The quality of the guess depends on the number of generated samples and the chosen distance. We use generated data of the same sample size as the training data (500). An ideal value for the distance is not known to the attacker. Therefore, we report the outcome for multiple values.

### Comparing DBMs with other deep generative models and multivariate imputation

In addition to DBMs, we also consider variational autoencoders (VAEs) [29] and generative adversarial networks (GANs) [30] as state-of-the-art generative models for comparison. Both models are, in contrast to the DBM, feed-forward neural networks and can be trained with a backpropagation algorithm [31]. Although the gradients are computed differently, DBMs, VAEs and GANs are all optimised via variants of gradient descent. This means that the number of training epochs and the learning rate are important hyperparameters. We account for this in our experimental setup by choosing a sufficiently small learning rate and evaluate the models at each epoch, finally choosing the model with the best performance. For the implementation of the training of VAEs and GANs, the model architectures, and the choice of the hyperparameters, we rely on Nußberger et al. [11], where we evaluated approaches on similar data. The complete code is available from GitHub [32] and employs the Julia package “Flux” [33] for building and training the networks.

We additionally use the MICE method (Multivariate Imputation by Chained Equations) via logistic regression. The algorithm for computing such a generative MICE model is as follows [8]:

1. Define a (random) order of the variables.
2. Estimate *p*(*v*_1_). For binary variables, this means simply calculating the frequency.
3. Calculate logistic regression models *R*_2_, …, *R*_*n*_ with independent variables *v*_1_, …, *v*_*n* − 1_ and dependent variable *v*_*n*_. (In our implementation, the variables are added consecutively to the model. If adding a variable leads to collinearity in the model, the variable is left out in this model and in all further models as independent variable. Also, if *v*_*n*_ is constant, this constant is simply used as prediction without fitting a regression model.)

Then data can be generated using this model by at first sampling ${\overset{\sim }{v}}_1$ according to *p*(*v*_1_) and then iteratively sample $\tilde{v}_{n}$ according to the probability predicted by regression model *R*_*n*_ using ${\overset{\sim }{v}}_1,\dots, \tilde{v}_{{n}-1}$.

To have a measure for a baseline performance, we use the method of independent marginals (IM) [8]. It uses only the estimated mean probability of each variable in the data set to generate synthetic data that are independent in all of the variables. In particular, for Bernoulli distributed, binary variables, a value of 1 is generated with a probability equivalent to the frequency in the empirically observed data.

## Results

As exemplary applications, we consider genetic variant data, so-called SNPs (single nucleotide polymorphisms). One main goal of deep learning on genetic data is to uncover interactions between genetic mutations that lead to certain (pathological) phenotypes. Particularly interesting are cases with many interacting mutations that are jointly responsible for a resulting phenotype. These cases are hard to detect with univariate testing of SNPs, which is used in genome-wide association studies (GWAS).

The analysis of genetic data is a relevant scenario for distributed privacy-preserving analyses because genetic data contains highly sensitive information about individuals, which cannot be shared easily across sites. Moreover, it is still expensive to produce this kind of data, so relatively few samples exist for many research questions. Therefore, this is a good use case for DBMs, which have been proven to be useful in this setting [10, 11].

### Example using simulated SNP data

**Fig. 4:**
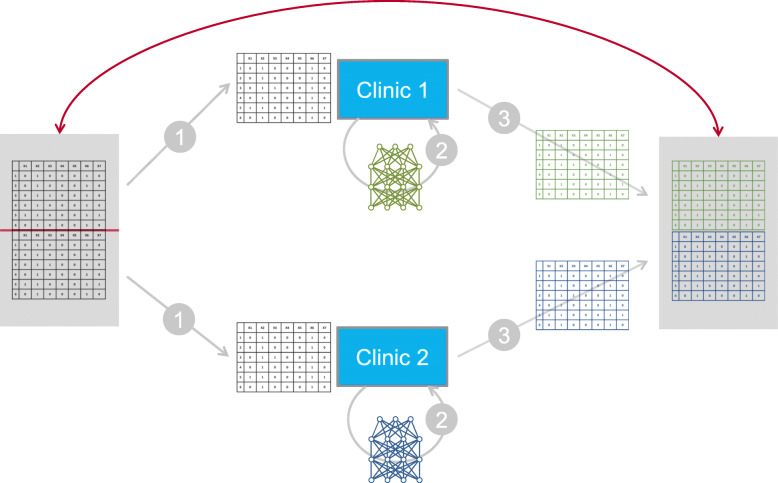
Sketch of the experimental setup for the comparison of original and generated data. In the first step, the original data is split into a number of smaller data sets, which are distributed in equal shares, consisting of consecutive parts of the data set, to the virtual sites. (For simplicity, only two sites/clinics are shown.) In step 2, separate generative models are trained at each site on their share of the data. In step 3, synthetic data are generated by each of the models and compiled to again form one overall data set. This synthetic data set will be visually compared to the original data set. For the results, see Fig. 5 below

Firstly, we conducted an experiment with simulated data to show that is it possible to learn and reproduce higher-level patterns employing DBMs in SNP-like data sets and evaluated their performance as generative models in this setting, showing the effect of the available sample size in particular (see Fig. 4). The artificial data set for our experiment consisted of binary data mostly consisting of zeros with some noise added from a Bernoulli distribution with a probability of 0.1. There are 50 SNP variables in total. The 500 samples are split equally into “cases” and “controls”. The “cases” have groups of five ones at five possible “SNP sets” among the 50 SNP variables. Such a SNP set, consisting of five SNP variables, could correspond to mutations that may deactivate a certain pathway, when they occur together.

In this experiment the randomly generated data set is split equally onto a number of virtual sites, where models are trained and then used to generate new data. This new data can then be visually compared with the original data (see Fig. 4).

**Fig. 5:**
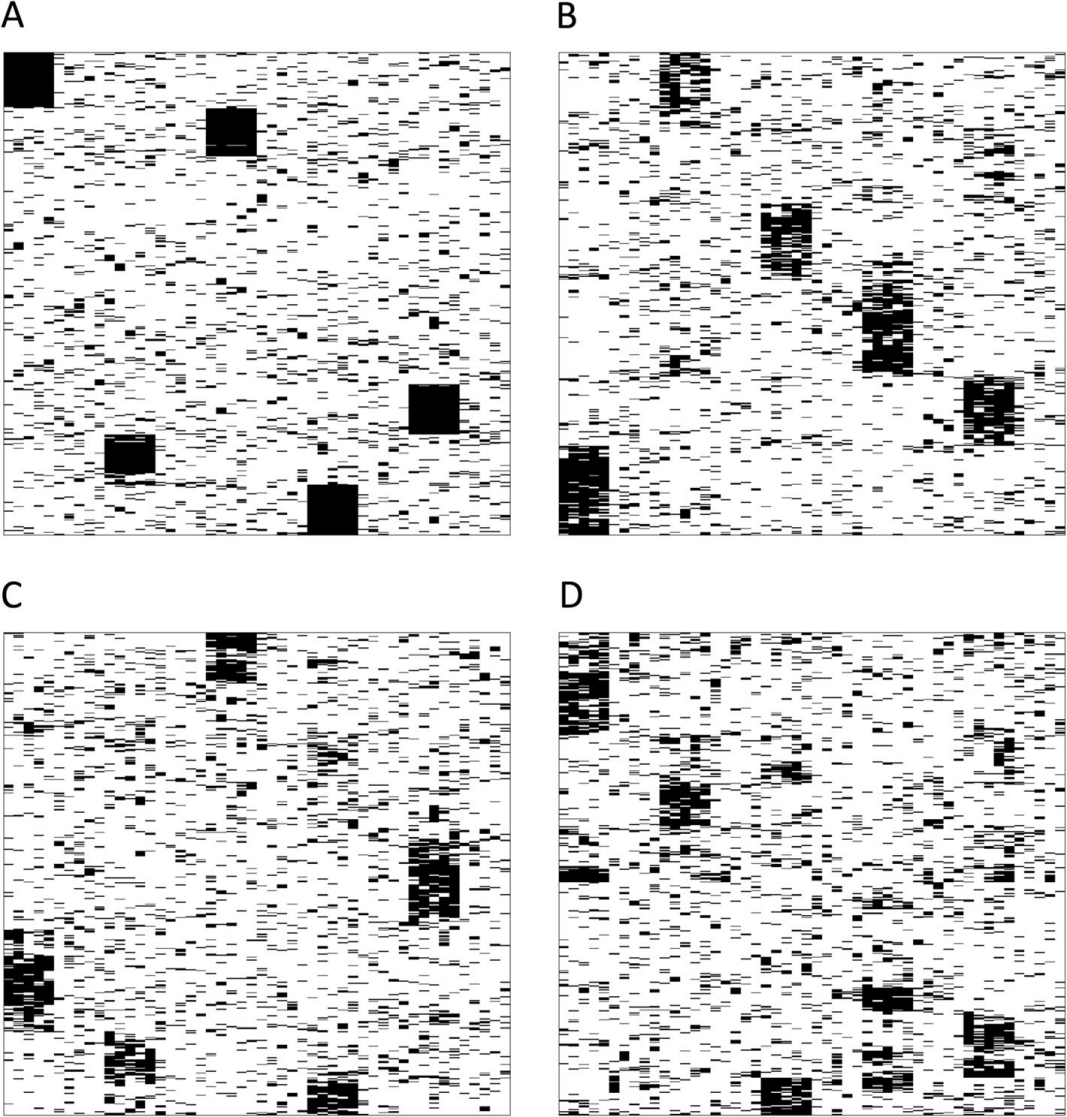
Hierarchical clustering view of a data set and associated synthetic data sets. The rows are the patients and the columns are the variables. The rows are clustered hierarchically [34]. Panel a shows the original data set, panel b shows data generated from one DBM that has been trained on the original data. Panels c and d show outputs of the experiment conducted with 2 and 20 sites, respectively. The SNP sets with the five consecutive 1s appear as black blocks in the hierarchical clustering view. The vertical positions of the black blocks change across the different sub plots because the noise in the other variables also influences the clustering. The horizontal position of the blocks, which is determined by the position of the genetic features, is the same in all four plots

The results of the experiment are shown in Fig. 5. The higher-level patterns, which here are the arrays of co-occurring SNPs, are preserved in the synthetic data, even in the case of 20 sites having only 25 patients. However, one can observe that the noise in the sampling output increases as the same amount of samples is distributed among a growing number of sites. Further, it is notable that there is some price to pay for using synthetic data since the output is not exactly the same as the input (see Fig. 5, e.g. comparing panels A and B).

For training the DBMs, we used two hidden layers comprising 50 and 10 nodes. We pre-trained the DBMs for 30 epochs with a learning rate of 0.001 and fine-tuned them with another 30 epochs and a learning rate of 0.1. To provide a rough idea of the time needed to train DBMs with our software, we measured the execution time, using the data set with 50 variables and 500 samples on a standard desktop computer with an Intel Core i5–4570 processor with 3.2 GHz, and running the DataSHIELD server in a virtual machine with only 1 CPU. The fitting of the DBM without monitoring took 1.7 s. The training took about 5.8 s with monitoring performed on the training data set and a test data set of 100 samples. (The times were measured after a first training run in the session to eliminate the precompilation time of the code in Julia.) The training time scales linearly with the number of samples, and it scales quadratically with the number of variables, if the number of hidden nodes is adjusted proportionally. With the number of samples scaled up to 2500, the training time became 11 s with monitoring and 5.2 s without monitoring for the data set with 50 variables. For 2500 samples, 500 variables, and two hidden layers of 500 and 100 hidden nodes we measured 4.6 min with monitoring and 2.9 min without monitoring for the training time of the DBM.

### Application on real SNP data and comparison with other generative models

To evaluate the performance of distributed analysis of SNP data based on real data, we consider human SNP data as provided in the 1000 Genomes Project [35]. In total, 5008 chromosomes from 2504 individuals are available [36]. The 1000 Genomes data, which are also employed to impute missing loci in genome-wide association studies [37], were retrieved from a website provided for the IMPUTE software [38]. Since the data are presented in haploid form, they consist of binary vectors where ‘1’ and ‘0’ indicate the minor and major allele respectively.

We removed variants with a minor allele frequency (MAF) below 0.2 to avoid absent minor alleles in small sub-samples occurring in our small sample-size scenario. 30 loci on chromosome 6, each spanning 50 SNPs, were randomly selected and were further used to benchmark our approach. Each chromosome is considered one sample in our analysis.

**Fig. 6:**
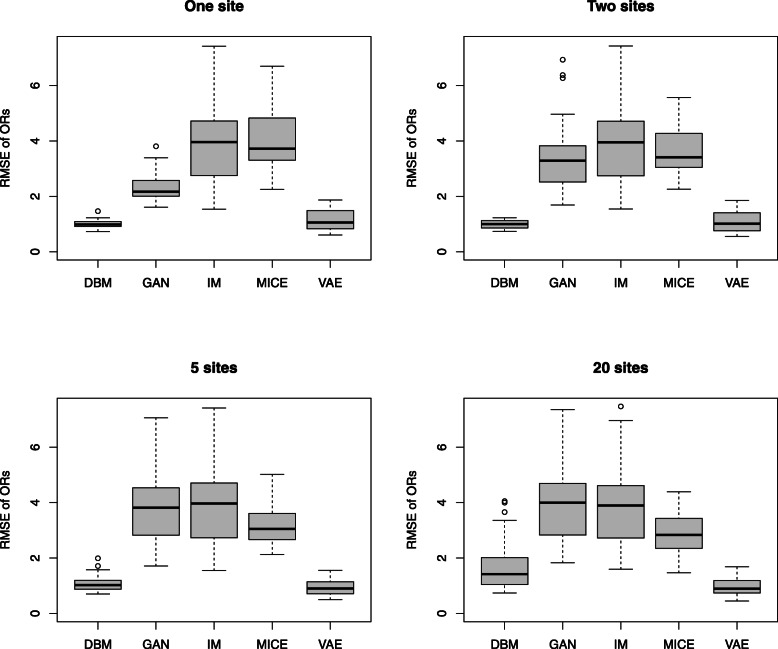
Performance comparison of the different model types based on odds ratios. The performance is quantified by the distance (rooted mean squared error) between log odds ratios computed from generated samples and validation data. Each model is evaluated on the same 30 different data sets. Each of the 30 data sets contains genetic variation data from 50 SNPs from randomly selected genetic locations. As shown in Fig. 4, the original data sets with 500 samples (chromosomes) are equally split into two, five and 20 sites, respectively. Results are shown for the combined generated data sets collected from the sites

**Fig. 7:**
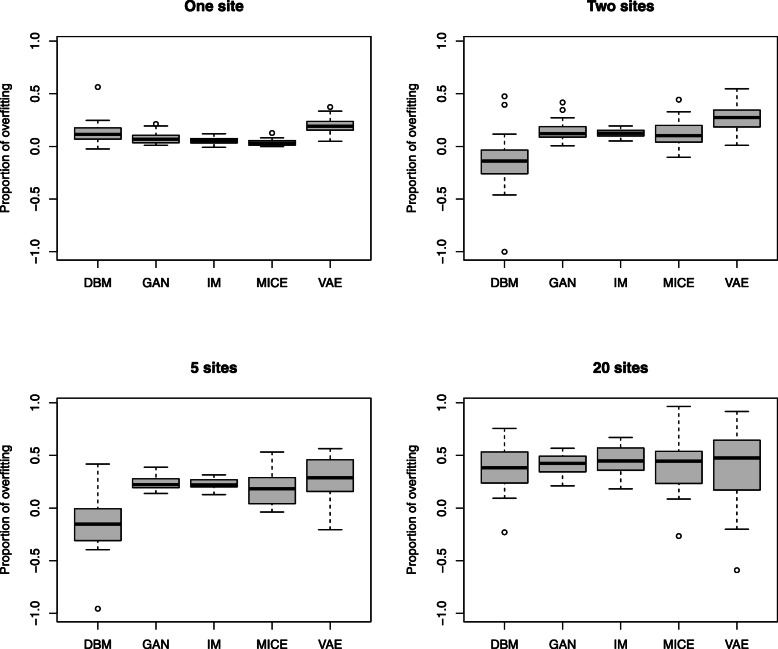
Proportions of overfitting in the models. Overfitting is indicated by a reduction in the distance of the log odds between generated data and the training data relative to the validation data. (See formula (1) in the methods section for a formal definition.) Positive values indicate overfitting, while negative values indicate that the approach performed actually better on the validation data than on the training data. All data points shown relate to the same data and model configurations that produced the results in Fig. 6

**Fig. 8:**
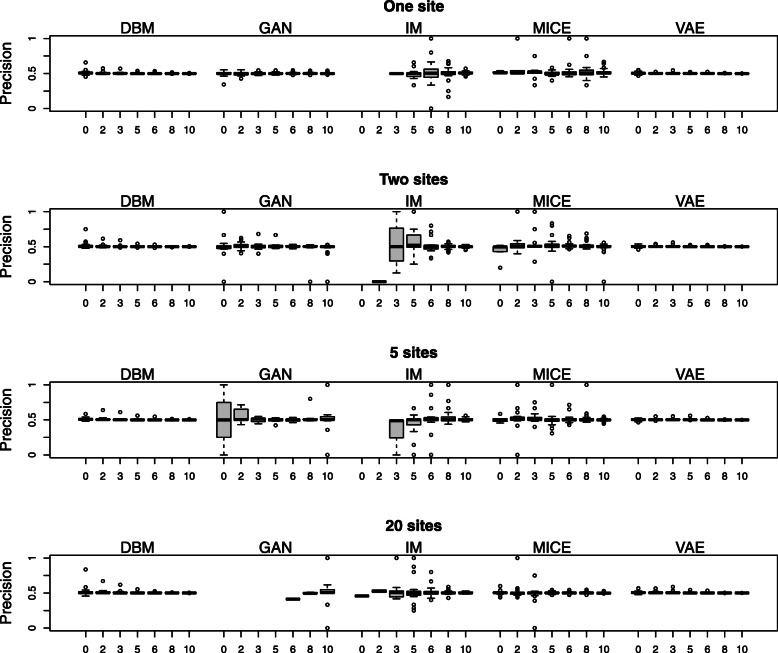
Precisions of distance-based membership attacks. The numbers on the x-axes indicate the Hamming distances. All data points correspond to the same data and model configurations that produced the results in Fig. 6

**Fig. 9:**
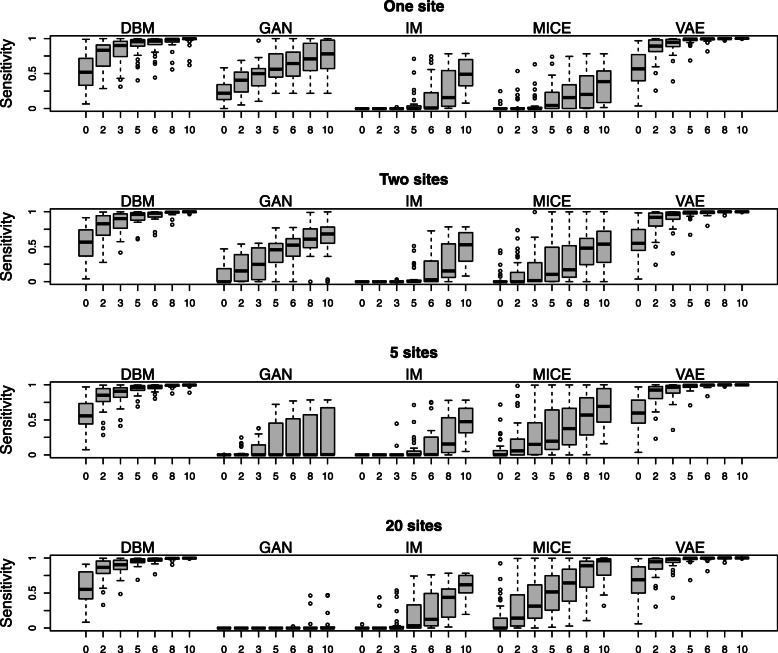
Sensitivities of distance-based membership attacks. Same as in Fig. 8, the numbers on the x-axes indicate the Hamming distances, and all data points correspond to the same data and model configurations that produced the results in Fig. 6

To compare the performance of DBMs with GANs, VAEs and MICE on this data, we used an experimental setup similar to the one shown in Fig. 4. We split each of the 30 data sets from the 30 different loci into a training data set *x*_*train*_ with 500 samples, a test data set *x*_*test*_ with 100 samples and a validation data set *x*_*val*_ with 1000 samples. These data sets are non-overlapping random subsamples of the original data set. The training data set is divided equally across the sites. As shown in Fig. 4., the generated data *x*_*gen*_ is obtained by combining the generated data from the models on the different sites, which have been trained on their share of the training data. Hyperparameter optimization is performed with respect to the RMSE between the log-odds in the generated data and the test data *d*(*x*_*gen*_, *x*_*test*_). Subject to hyperparameter optimization are the range of training epochs (up to 2000) and the initialization of the model parameters (15 different random initializations). Only the set of models with the best performance, i.e. the lowest distance of log odds in the validation set and the generated combined data set, is shown, resulting in one data point for each combination of data set and model type. The summary of these results is shown in Fig. 6, Fig. 7, Fig. 8, Fig. 9, and Table 3.

The architectures for the models are chosen as in Nußberger et al. [11], with a similar number of hidden nodes in the different models. The code for the experiment is also adapted from there and can be found on GitHub [32], together with the data. The code is written entirely in Julia, without using DataSHIELD, as there are no corresponding packages for the other approaches in DataSHIELD yet. Running the complete experiment for producing the plot took 25 h on a cluster of three computers with 8, 12, and 28 cores with clock speeds of about 3 GHz and at least than 8 GB RAM per core.

In the results of the experiment shown in Fig. 6 and Table 3, we can see that both DBMs and VAEs significantly outperform the GAN and the MICE approach in terms of the generative performance, measured by the RMSE of the log odds ratios. DBMs and VAEs show a comparable performance. With respect to the proportion of overfitting (see Fig. 7 and Table 3), the DBM is comparable or better than the VAE in our scenario in all sample sizes. In the scenario with one site and 500 samples, the DBM and the other neural networks overfit slightly more than the MICE method. In the settings with up to 250 samples per site, the DBM exhibits less overfitting than MICE. This is remarkable as it indicates that the synthetic data generated by the DBM can generalize the structure from the training data equally well as sequences of logistic regression models fitted on the data. In the extreme setting with 20 sites, i.e. only 25 samples and 50 variables, we can observe that overfitting becomes very strong in all kinds of approaches, which probably would result in the disclosure of too much information about the training data.

A higher disclosure risk for the more complex models cannot be seen in the results of the membership attack (see Fig. 8 and Fig. 9). The precision of detecting whether a sample is a training sample is very near to 0.5, i.e., a random guess, for DBMs and VAEs as well as for GANs, IM, and MICE (see Fig. 8). With an increasing number of sites, the precisions of attacking GANs and IM get unstable but are generally centred around 0.5 as well. In contrast, the precisions for DBMs and VAEs are very stable across varying sample sizes and numbers of sites with very few outliers.

For DBMs and VAEs, the sensitivity of the membership attack (see Fig. 9) is generally high and only slightly affected by the number of sites. For GANs, IM, and MICE, however, the sensitivity is relatively poor. The decrease of the sensitivities for the GANs with an increasing number of sites (and samples per site) points to the fact that fitting the GANs becomes increasingly harder with smaller sample size. More generally, the sensitivity can also be regarded as a measure for the performance on the training set because it measures how near generated samples are to training samples on the whole. If there are only few true positives and false positives, i.e., if the generated samples are mostly far away from either training or test samples, the variance of the precision gets higher. The higher variability in the precision of GANs, IM and MICE can therefore be explained by their worse overall performance as generative models in this setting.

The results for DBMs and VAEs for the sensitivity are also in concordance with their behaviour concerning the RMSE of odds ratios as performance criterion: While DBMs and VAEs display a very similar performance regarding the performance on the validation data, VAEs have a slightly higher sensitivity in the settings with one to five sites. This indicates a slightly higher performance for VAEs on the training set, which is in line with a higher amount of overfitting.

## Discussion

While synthetic data are a promising option for enabling a broad set of statistical analyses in a distributed setting, an accessible implementation is currently lacking. We described an extension of the popular DataSHIELD framework for distributed analysis under data protection constraints. In particular, we leveraged generative deep learning for obtaining synthetic data. To make these algorithms available in DataSHIELD, we established a connection from the R environment, which is the basis of DataSHIELD, to the Julia language, which is better suited for implementing deep learning algorithms. To the user, this complexity is masked via a convenient R package.

We chose deep Boltzmann machines (DBMs) as generative models to be implemented in DataSHIELD because of their advantages in certain use cases. As shown in our feasibility study, DBMs can deal with a low number of training samples and are hard to overfit. This makes them especially suitable for distributed settings with small data sets at each of the sites. Nevertheless, it is possible to train the DBMs also on data sets with a large number of samples as the training time scales linearly with the number of samples. Another area in which DBMs excel is conditional sampling, which can be implemented in a straightforward way using Gibbs sampling. A possible application of conditional sampling is using a DBM trained on gene expression data, to simulate the up-regulation of one pathway and observe changes in the expression of other genes. Another example could be to generate data for medication or comorbidities conditioned on diagnoses in data from electronic health records.

In recent years, other generative models, most importantly generative adversarial networks (GANs) [30] and variational autoencoders (VAEs) [29, 39], have become popular and have been proven to work well, particularly on large data sets. In contrast to deep Boltzmann machines, which rely on Markov Chain Monte Carlo methods to be trained, GANs and VAEs are trained using backpropagation of errors, requiring a different implementation approach for the training. Anyway, the methodology of training generative models at the different sites, returning only learning curves and synthetic data via the DataSHIELD infrastructure, as done in our implementation with DBMs, can be extended to all types of generative neural network models. For models that are not directly available in R, wrapping techniques can be used to make them available in DataSHIELD, as demonstrated here with the JuliaConnectoR for connecting R to Julia. Due to the good performance of VAEs, we also plan to implement VAEs as generative models in DataSHIELD, and to combine the different types of approaches to minimize the bias in generated data.

The results of our empirical investigation with simulated data showed that some structure is maintained even with very small sample sizes, but performance could potentially still be improved. For example, partitioning of layers can be used to further decrease the number of samples needed to find informative structure in the data [10]. The visual inspection of the samples in the hierarchical clustering shows the utility of the generated data for clustering. The performance of the DBM with respect to the error in the odds ratios indicates also the usability of the generated data for performing other types of analyses, such as logistic regression, where the odds ratio is used as a score to describe the effect of one variable on another.

The results of the simulated membership attack do not show a higher disclosure risk for DBMs and VAEs compared to the simpler methods MICE and IM. We also provide another disclosure analysis based on the degree of overfitting of the studied approaches. Compared to the differential privacy measure *ε* and the simulated membership attack, this approach does not rely on a potentially arbitrary threshold and instead directly indicates if a model has a tendency to focus on highly specific details, i.e. to copy the data, which could easily be exploited for identifying individuals based on synthetic data. In fact, quantifying overfitting has already been employed to detect data copying by generative models used for generating synthetic images [40]. Based on the degree of overfitting and the simulated membership attack as disclosure analyses, we show that the DBM does not leak more information about the individuals in the training data than could be extracted via a sequence of logistic regression models such as in the MICE method. It can be argued that this may suffice, since logistic regression results are also allowed to be communicated in DataSHIELD [41]. Therefore, this level of data protection may suffice if the data is only analysed by a defined user group that agreed to a certain code of conduct. This may apply to the use of DataSHIELD in a defined research context. Yet, for data that is to be released to the public without any restrictions, there probably need to be stricter requirements for privacy. Asserting absolute levels of privacy such as *ε*-differential privacy with a certain *ε* would certainly be desirable in such a case, even if the value of *ε* is still an open topic and there is no standard value for it such as, e.g., for the 0.05 significance level.

## Conclusions

With the presented extension to the DataSHIELD software, we add the possibility of generating artificial data sets that preserve the higher level patterns from individual patient data that may be distributed among different sites. These generated data sets can then be analysed to extract patterns from the original data without access to individual patient data. The results presented here indicate that our proposed approach is ready for use in real world applications. This is facilitated by the user-friendly design of our implementation complemented by extensive documentation. More generally, the proposed implementation provides a sound basis for subsequent extensions to other generative approaches for synthetic data in distributed analysis.

## Data Availability

The analysis based on simulated data is fully described in the article. The accompanying software is available from GitHub [21, 22]. The original data for the method comparison on real SNP data can be found on a website provided for the IMPUTE software (https://mathgen.stats.ox.ac.uk/impute/1000GP_Phase3.html). The pre-processed data from the randomly selected loci and chromosomes can be found on the GitHub repository that also contains the complete reproduction script for the experiment (https://github.com/stefan-m-lenz/dist-gen-comp/).

## Abbreviations

AIS: annealed importance sampling
CPU: central processing unit
DBM: Deep Boltzmann machine
DBN: Deep belief network
GAN: Generative adversarial network
GWAS: Genome-wide association study
IM: Independent marginals
MICE: Multivariate imputation by chained equations
RBM: Restricted Boltzmann machine
SNP: Single nucleotide polymorphism
VAE: Variational autoencoder
